# Gene Expression from Bronchoscopy Obtained Tumour Samples as a Predictor of Outcome in Advanced Inoperable Lung Cancer

**DOI:** 10.1371/journal.pone.0041379

**Published:** 2012-07-27

**Authors:** Rafal Suwinski, Artur Klusek, Tomasz Tyszkiewicz, Maria Kowalska, Bogna Szczesniak-Klusek, Marzena Gawkowska-Suwinska, Andrzej Tukiendorf, Jerzy Kozielski, Michal Jarzab

**Affiliations:** 1 Radiotherapy Clinic and Teaching Hospital, M. Sklodowska-Curie Memorial Cancer Centre and Institute of Oncology, Gliwice, Poland; 2 Department of Pulmonology, General Hospital, Chorzow, Poland; 3 Department of Nuclear Medicine and Clinical Endocrinology, M. Sklodowska-Curie Memorial Cancer Centre and Institute of Oncology, Gliwice, Poland; 4 Department of Pathology, M. Sklodowska-Curie Memorial Cancer Centre and Institute of Oncology, Gliwice, Poland; 5 Department of Biostatistics, M. Sklodowska-Curie Memorial Cancer Centre and Institute of Oncology, Gliwice, Poland; 6 Pumonology Clinic and Teaching Hospital, Silesian Medical University, Zabrze, Poland; University of Barcelona, Spain

## Abstract

**Background:**

Several studies have shown the prognostic and predictive potential of molecular markers in combined therapy for lung cancer. Most of them referred, however, to operable early stage NSCLC. The aim of the present study is to correlate the expression of multiple mRNA markers in bronchoscopy obtained cancer specimens with clinical outcome of advanced lung cancer.

**Methods:**

Bronchoscopy cancer specimens were taken from 123 patients with radiological diagnosis of advanced lung tumor. Out of 123 patients 50 were diagnosed with squamous cell cancer, 17 with adenocarcinoma, 12 with NOS, 32 with SCLC and one with large cell neuroendocrinal cancer. In 11 patients other tumours were diagnosed. The group was heterogeneous with respect to clinical stage, performance of the patients and treatment. Quantitative real time PCR was carried out by ABI 7900 HT machine, with Universal Probe Library (Roche) fluorescent probes. The genes selected for the analysis were ERCC1, EGFR, BRCA1, CSF1, CA9, DUSP6, STAT1, ERBB3, MMD, FN1, and CDKN1B.

**Results:**

More than 50 ng of RNA (the amount considered sufficient for the analysis) was isolated in 82 out of 112 lung cancer specimens (73%), including 60/80 (75.0%) of NSCLC specimens and 22/32 (68,7%) of SCLC samples. The highest Cohen’s κ coefficient for discrimination between small cell, squamous cell and adenocarcinoma was found for CDKN1B, CSF and EGFR1 (κ = 0.177, p = 0.0041). A multivariate Cox regression model has shown a significant impact of clinical stage (p<0.001, RR = 4.19), ERCC1 (p = 0.01, RR = 0.43) and CA9 (p = 0.03, RR = 2.11) expression on overall survival in a group of 60 patients with NSCLC.

**Conclusion:**

These results show the feasibility of multiple gene expression analysis in bronchoscopy obtained cancer specimens as prognostic markers in radiotherapy and chemotherapy for advanced lung cancer. A limiting factor was relatively high proportion of samples from which sufficient amount of RNA could not be isolated.

## Introduction

Several studies demonstrated the potential of gene expression profiles for prediction of outcome in non-small cell lung cancer (NSCLC) [1]–[8]. Most of these studies referred, however, to surgically treated early stage disease. The accessibility and quality of tumour samples obtained at surgery makes this approach feasible, thus various prognostic signatures for early stage disease were proposed.

Unfortunately, lung tumours are frequently diagnosed in advanced clinical stage, in which radiotherapy and/or chemotherapy are chief treatment options. Also, the pathological diagnosis of small cell lung cancer (SCLC) precludes use of surgery in most of the cases. Consequently, the prognostic signatures that refer to the patients treated with surgery may have limited, if any, applicability to individuals and datasets in which chemotherapy and/or chemotherapy is the principal therapeutic option. One may expect that the same genes that predispose tumour cells to poor differentiation, thus to propensity for dissemination after surgery, may entail enhanced radiosensitivity and/or chemosensitivity due to short cell turnover. Such distinctions emphasize the demand for the assays that evaluate gene expression from bronchoscopy obtained tumour specimens, and relate the expression to the clinical outcome. The development of prognostic signatures for advanced inoperable tumours has lagged, however, behind similar attempts with surgically obtained specimens [9]. This created the basis for the present study.

## Methods

### Patients

Selection criteria included patients with radiologically diagnosed lung tumour that was suspected for malignancy. Only the patients with bronchoscopy accessible advanced tumours, deemed inoperable at the initial diagnosis, were considered. An informed consent was required for inclusion to the study. A protocol of this research was approved by the local Bioethical Committee according to national regulations.

Between November 2006 and April 2009 tumour samples were obtained during routine bronchoscopy in 123 patients. Samples were appraised immediately after the biopsy and part of the specimen was used for pathological examination, while the remaining part was stored for gene expression analysis.

Out of 123 patients 50 (40.6%) were diagnosed with squamous cell cancer, 17 (13.8%) with adenocarcinoma, 12 (9.7%) with NOS (not otherwise specified) NSCLC, 32 (26.0%) with small-cell lung cancer and one with large cell neuroendocrinal cancer. In 7 patients other tumour types were diagnosed, including 2 cases of lymphoma and 5 metastatic tumours. In 2 patients tumour did not appear to be malignant, and in 3 cases the definitive pathological diagnosis could not be established.

In summary 112 out of 123 samples (91.1%) were used in the study. This included 80 samples of non-small cell lung cancer, and 32 of small cell lung cancer.

### Gene Expression Analysis

Tumour samples were placed in 5 ml of RNA later and stored in −20°C before the analysis. Isolation of RNA was performed with RNeasy Midi Kit (Qiagen) following standard procedures. Purified RNA was stored in −70°C. The concentration of RNA was assessed by spectrophotometry (260 nm absorption) using NanoDrop ND-1000. Quality of RNA was assessed by capillary electrophoresis in Bioanalyzer 2100 (Agilent Technologies) using RNA 6000 Nano Assay. A degree of RNA degradation was assessed by estimation of RNA Integrity Number (RIN). cDNA was synthesised on the RNA matrix by reverse transcription using Omniscript Kit (Qiagen).

Quantitative real time PCR was carried out by ABI 7900 HT machine, with Universal Probe Library (Roche). The expression of target genes was normalized relative to six reference genes (UBE2D2, PGK1, HADHA, EIF3S10, CCT7 and B2M).

Eleven genes were selected for the analysis: ERCC1, EGFR, BRCA1, CSF1, CA9, DUSP6, STAT1, ERBB3, MMD, FN1, and CDKN1B. Such selection was based on the published prognostic signatures, most of which referred to surgical series of patients with NSCLC. Table 1 summarizes the probable mechanism of action of these genes, as well as the expected outcome related to over-expression of the gene. We note that the expected outcome refers, predominantly, to early stage tumors, and not to the patients with advanced inoperable disease.

**Table 1:** The probable mechanism of action of selected genes and the expected outcome related to over-expression in early stage lung cancer.

Gene	Mechanism of action	Postulated effect of overexpression In earlystage disease	References
Breast cancer 1 - **BRCA1**	Helps repair damaged DNA	Worse prognosis	Rosel et al. [12]
Carbonic anhydrase 9 (**CA9**)	Involved in cell proliferation and transformation, a surrogate marker of tumor hypoxia	Worse prognosis	Swinson et al. [13]
Cyclin-dependent kinase inhibitor1B (**CDKN1B**)	A cell cycle inhibitor	Better prognosis	Shapiro GI et al. [14]
Colony stimulating factor 1 (**CSF1**)	Growth factor involved in the proliferation, differentiation, and surival of monocytes,macrophages	Worse prognosis	Skrzypski et al. [2]
Dual specificity phosphatase 6 (**DUSP6**)	Negatively regulates members MAP kinasesuperfamily which are associated with cellularproliferation and differentiation.	Worse prognosis	Chen et al. [1]
Epidermal growth factor receptor (**EGFR**)	Initiates signal transduction cascades leading toDNA synthesis and cell proliferation	Worse prognosis	Skrzypski et al. [2]
Receptor tyrosine-protein kinase (**erbB-3**)	Encodes a member of the epidermal growth factor receptor family of receptor tyrosine kinases, higher expression in non-smoking females	Disputed	Chen et al. [1], Kawano [15]
Excision repair cross-complementing rodent repairdeficiency -**ERCC1**	Nucleotide excision repair of damaged DNA	Better prognosis	Olaussen et al. [16]
Fibronectin **FN1**	Plays a major role in cell adhesion, growth,migration and differentiation	Worse prognosis	Han et al. [17]
**MMD** monocyte-to-macrophage differentiation associated protein	Expressed in mature macrophages. Macrophage activation may promote cancer metastasis	Worse prognosis	Chen et al. [1],
**STAT 1** Signal Transducer and Activator of Transcription 1	Involved in upregulating genes leading to anincreased expression of Interferon StimulatedGenes. Causes arrested growth and apoptosis inmany types of cancer cells	Better prognosis	Chen et al. [1],

### Statistical Analysis

The principal goal of the analysis was to categorize the tumours according to clinical prognosis, utilizing the data on gene expression. The group, however, was heterogeneous with respect to tumour pathology, clinical characteristics and treatment that could significantly affect the clinical outcome, irrespectively of gene expression. Therefore, the statistical methods allowed adjustment for the confounding clinical factors.

The influence of gene expression on survival was analyzed using univariate and multivariate Cox regression model. Normalized gene expression was dichotomized, such that it was coded as 0 (low) or 1 (high) using median expression value as a cut-off [2].Overall survival was calculated as the time from the diagnosis to death (uncensored) or as the time from the diagnosis to the last follow-up (censored). Considering exploratory purpose of this research and relatively small sample size, Cox f test was used to evaluate significance of differences in survival between two groups. This test is designed for analysis of small groups, unlike log-rank test that is used for the analysis of large clinical datasets.

A univariate Cox model of survival was used to calculate relative risk (RR) for death in dichotomized groups. Only the variables that appeared significant, or that showed a trend for significance (RR<0.7 or RR>1.3 and p<0.15) were considered in a multivariate model. Selection based on statistical trends is justified by clinical heterogeneity of the group that, whenever unaccounted, could confound the possible prognostic impact of gene expression. A multivariate Cox proportional hazard regression model identified the variables that significantly and independently influenced survival. The model was optimized using backward stepwise regression. The variables that significantly and independently influenced overall survival were used to construct a prognostic signature based on results of a multivariate study.

Since the clinical prognosis and the management of SCLC differ entirely from NSCLC survival in these two groups was analysed separately.

To identify genes that can discriminate between 3 major pathological sub-groups (squamous cell cancer, adenocarcinoma and small cell lung cancer) Student’s t-test was used at the onset. To further explore possible relationship between gene expression and pathological type of the tumour k-means method was used. This method represents the cluster analysis which aims in partition of n observations into k clusters in which each observation belongs to the cluster with the nearest mean [10]. The best association between expressions of genes and pathological type of the tumour was established using the Cohen’s κ coefficient which is a statistical measure of inter-rater agreement for categorical items [11]. We note that the genes analyzed in this study were selected as potential prognostic factors in cytotoxic therapy and not as the potential discriminators between the tumour types.

## Results

### Quality of RNA

More than 50 ng of RNA (the amount considered sufficient for the analysis) was isolated in 82 out of 112 lung cancer specimens (73%), including 60/80 (75.0%) of NSCLC specimens and 22/32 (68.7%) of SCLC samples. Among the patients with squamous cell cancer a sufficient amount of RNA was obtained in 38/50 (76.0%) compared to 12/17 (70.6%) with adenocarcinoma and to 10/12 (83.3%) with NOS NSCLC. Very good RNA integrity (RIN 8–10) was obtained in only 12.3% of the samples. These shows that the analysis of bronchoscopy obtained tumor specimens demands further optimization before clinical implementation. The analysis, as shown, was based on gene expression status evaluated in 82 specimens from whom sufficient amount of RNA was isolated (60 NSCLC and 22 SCLC).

### Gene Expression According to Pathology of the Tumor

Student’s t-test revealed that BRCA1, CDKN1B and CSF expression differed significantly between small-cell and non-small cell cancer (p-values 0.01, 0.02, and 0.02 respectively). Two other genes (EGFR1, STAT1) have shown a trend. (p values 0.07 and 0.09 respectively). By contrast, no significant differences in gene expression were found between squamous cell cancer and adenocarcinoma, although there was a trend for BRCA1, CDKN1B and ERCC1. The highest Cohen’s κ coefficient for discrimination between small cell, squamous cell and adenocarcinoma was found for CDKN1B, CSF and EGFR1 (κ = 0.177, p = 0.0041). Such estimate indicates a relatively small but significant association between expressions of these genes and pathological type of the tumour.

### Clinical Factors vs. Survival in the Target Group

Table 2 illustrates clinical characteristics of 60 patients with NSCLC who had known gene expression status. The group was heterogeneous with respect to clinical stage, performance of the patients and treatment. As expected, the majority of the patients were in advanced clinical stages, including 48% with stage IV disease. Only 2 individuals (3%) were in stage I-II disease (both were considered inoperable due to comorbidities). Sixty percent of the patients had chemotherapy (cisplatin based doublets), 57% had radiotherapy with total doses ranging from 20–70 Gy (median of 30 Gy). Median follow-up is 3.6 years).

**Table 2:** Clinical characteristics of 60 patients with advanced NSCLC and known gene expression status.

Variable		N (%)	2-year OS	RR	p-value
**Age**	<61 years	29 (48.3%)	18%	1	0.82
	≥61 years	31 (51.7%)	22%	0.94	
**Gender**	F	14 (23.3%)	43%	1	0.02
	M	46 (76.7%)	13%	2.4	
**Zubrod**	0–1	42 (70.0%)	30%	1	0.0007
	>1	18 (30.0%)	0%	2.9	
**Stage**	I-III	31 (51.7%)	33%	1	0.0002
	IV	29 (48.3%)	4%	3.6	
**RT**	Yes	35 (58.3%)	31%	1	0.005
	No	25 (41.7%)	4%	2.9	
**CT**	Yes	36 (60,0%)	25%	1	0.005
	No	24 (40,0%)	7%	2.4	

Stage, performance status and gender significantly influenced survival in a univariate model, but in a multivariate analysis only clinical stage appeared to have and independent prognostic significance (RR = 3.56, 95% CI = 1.95–6.45, p = 0.00003). Therefore, clinical stage was further considered as the major factor that may confound the possible prognostic significance of gene expression.

### Univariate Influence of Gene Expression on Survival

Table 3 demonstrates influence of 11 selected genes on overall survival in a group of 60 patients with NSCLC. Out of 11 genes only the expression of ERCC1 had a significant influence on survival, while the expressions of four other genes (CA9, ERBB3, FN1 and STAT1) showed a trend (RR<0.7 or RR>1.3 and p<0.015). High expression of 3 of these genes (CA9, FN1 and STAT1) was related to increased hazard of death, while over-expression of ERCC1 and ERBB3 had a protective effect. Most importantly (as will be further discussed) such outcome has a rational explanation in biological function of these genes, and in the outcome of the published studies. Weakness of significance of individual gene expression for survival is not surprising, considering clinical heterogeneity and small sample of the group.

**Table 3:** Influence of gene expression on overall survival in 60 patients with NSCLC (univariate analysis).

Gene	RR	95% CI	2-year OS (High vs. Low)*	p-value (Cox f test)
BRCA1	1,43	0,80–2,52	20% vs. 16%	0,19
CA9	1,61	0,90–2,84	28% vs. 13%	0,07
CDKN1B	1,01	0,93–1,09	23% vs. 12%	0,49
CSF1	1,17	0,85–1,59	19% vs. 17%	0,31
DUSP6	0,99	0,84–1,17	20% vs. 20%	0,45
EGFR	0,88	0,49–1,54	17% vs. 19%	0,36
ERBB3	0,68	0,38–1,19	13% vs. 23%	0,09
ERCC1	0,59	0,33–1,04	29% vs. 14%	0,03
FN1	1,44	0,81–2,56	13% vs. 27%	0,14
MMD	0,86	0,48–1,51	23% vs. 20%	0,22
STAT1	1,40	0,77–2,50	13% vs. 33%	0,15

* 2 year survival for high (H) and low (L) gene expression respectively significant differences and trends (p≤0.15) are underlined.

**Figure 1:**
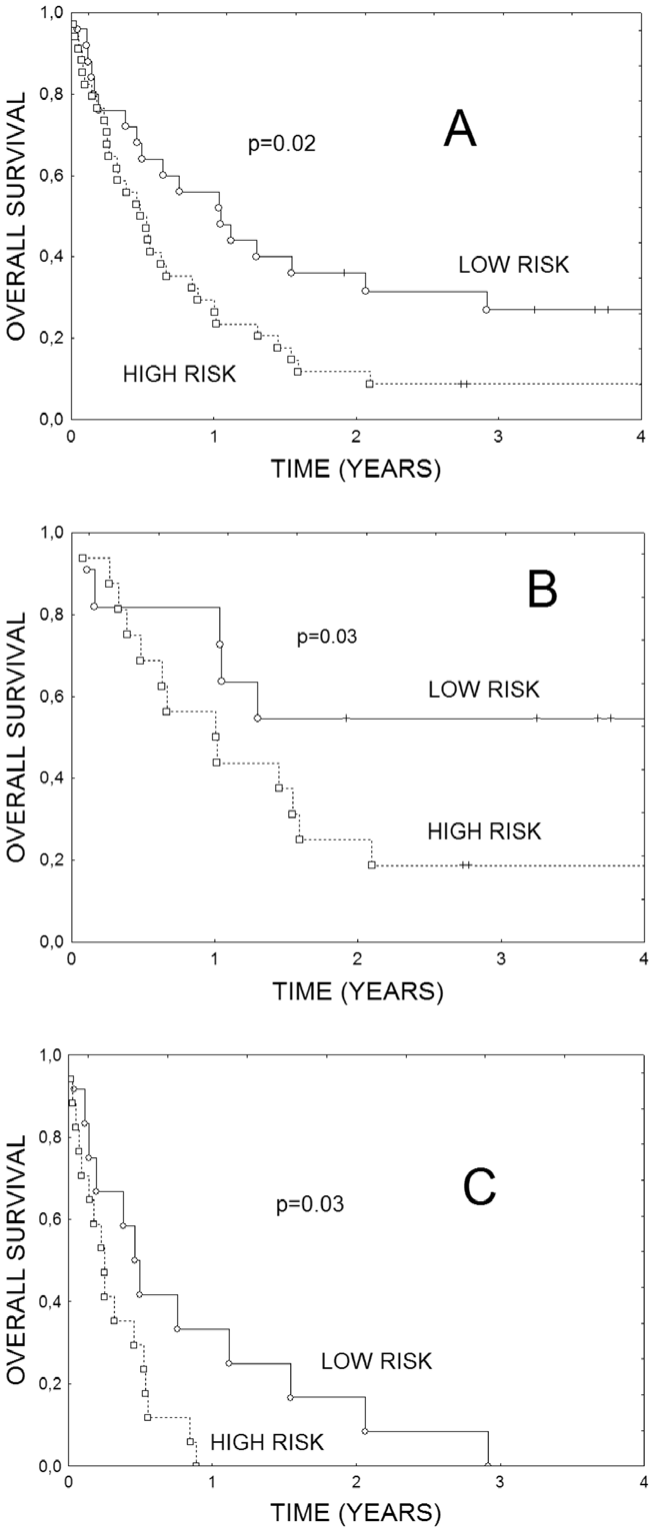
Overall survival of the patients with NSCLC according to a 5-gene signature (over-expression of CA9, FN1, STAT1 was coded as +1, over-expression of ERCC1 or ERBB3 was coded as −1) Total score ≥0 denoted high risk. A) 60 patients with NSCLC. B) 31 patients with localized disease (stage I-III). C) 29 patients with metastatic diseases (stage IV).

For the exploratory purposes we constructed a prognostic score in which overexpression of each risk genes (CA9, FN1 or STAT1) was coded as +1, while over-expression of each of the protective gene (ERCC1 or ERBB3) was coded as −1. The scores were added. The sum below zero denoted low risk, while the sum of zero or more denoted high risk. Figure 1a illustrates overall survival according to such five-gene signature. The difference in survival between high and low risk groups was significant (RR = 1.89, p = 0.02). When the analysis was restricted to 31 patients with localized disease the difference between high and low risk groups remained significant (RR = 2.3, p = 0.03, Figure 1b). It also remained significant in 29 patients with metastatic disease (RR = 2.3, p = 0.03, Figure 1c).

### Multivariate Analysis

Five genes that appeared significant or demonstrated trend in a univariate model were included in a multivariate analysis (ERCC1, ERBB3, CA9, FN1 and STAT1). Such analysis revealed that 3 out of 5 genes (ERBB3, FN1 and STAT1) cannot be considered as independent prognostic factors. The overexpression of ERBB3 was more common in females, and was thus related to stage of disease (advanced stage was more common in males). Likewise, the overexpression of FN1 was more common in the most advanced clinical stages. The overexpression of STAT1 was correlated with overexpression of CA9. Two genes remained significant in variant of a multivariate model that did not account for tumor stage: ERCC1 (RR = 0.53, 95% CI 0.30–0.96, p = 0.03) and CA9 (RR = 1.79, 95% CI 1.00–3.21, p = 0.05).

**Figure 2:**
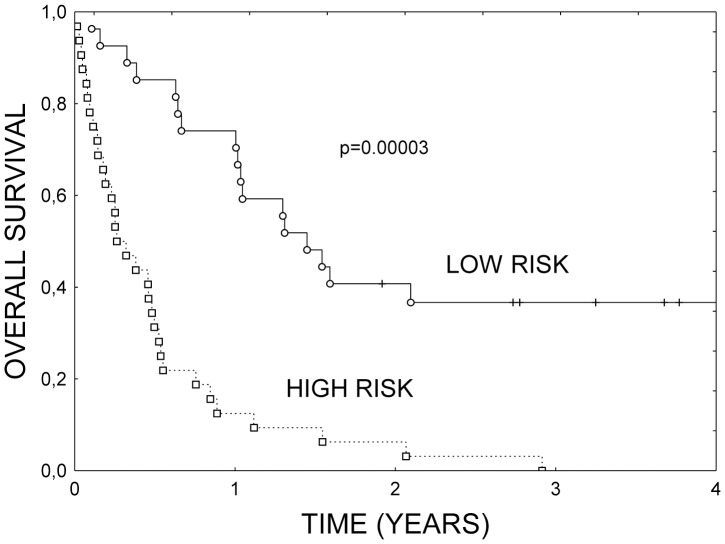
Overall survival of 60 patients with NSCLC according to the proposed risk score, R = (4.19×Stage)+(2.11×CA9)−(0.42×ERCC1). Values above 2.5 denoted high risk.

Multivariate model that accounted also for the stage of disease confirmed a significant and independent prognostic value of ERCC1 and CA9 expression (Table 4). The prognostic score constructed based on results of the multivariate analysis was as follows:


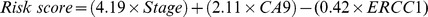


where clinical stage, as well as CA9 and ECCC1 expressions were dichotomized and coded as 0 or 1. Values above the median of 2.5 denoted high risk. Figure 2 illustrates survival curves according to risk groups. The difference in survival between the groups was highly significant (RR = 4.40, 95% CI 2.35–8.17, p = 0.00003), and the difference surpassed the univariate influence of stage (RR = 3.56).

**Table 4:** Influence of clinical stage and gene expression on overall survival in 60 patients with NSCLC (multivariate analysis).

Gene	RR	95% CI	p-value
STAGE	4.19	2.21–7.89	0.0000009
ERCC1	0,43	0.22–0.84	0.01
CA9	2.11	1.08–4.12	0.03

### Small Cell Lung Cancer

Out of 22 patients with SCLC 7 had limited and 15 extensive disease. The extent of disease did not appear to significantly influence survival (RR-0.95, p = 0.93). By contrast, over-expression of 2 genes appeared to significantly decrease survival (CA9: RR = 1.6, p = 0.04 and MMD, RR = 1.8, p = 0.03). The clinical implication of this finding is, nevertheless, questionable due to very small size and clinical heterogeneity of the group.

## Discussion

Gene expression has been successfully used for classification and prognosis of non-small cell lung cancer, although recent review of the published data suggest that this approach is not yet ready for clinical application [6]. Most of the relevant studies referred to surgical series [1]–[8], [12]–[17], with only few that attempted to analyze gene expression profiles based on bronchoscopy obtained samples [9]. By contrast, evaluation of EGFR mutations as predictive assay for tyrosine kinase inhibitors in advanced adenocarcinoma of the lung was considered a major breakthrough, and emerged as a new diagnostic tool [18]. This demonstrates that, in spite of difficulties and limitations, molecular markers will likely play an increasing role in diagnostics, prognosis and prediction for advanced non-small cell lung cancer.

The results of the present study indicate that the analysis of gene expression profiles based on bronchoscopy obtained samples is feasible, but the methods that were used demand optimization. The major limiting factor was relatively high proportion of samples from which sufficient amount of RNA could not be isolated. Another factor that contributed for undesirable drop-out rate of the cases was the pathological diagnosis that had not been available before the bronchoscopy. A proportion of the patients had, thus, different diagnosis from that anticipated based on symptoms, clinical examination and imaging.

Out of eleven genes studied high expression of 3 genes (CA9, FN1 and STAT1) was related to increased hazard of death, while over-expression of ERCC1 and ERBB3 had a protective effect. Such outcome has a rational support in biological function of these genes, and in the outcome of selected published studies.

ERCC1 appeared to be most strongly related to the prognosis in the present study. It is responsible for nucleotide excision repair of damaged DNA. Over-expression of this gene may enhance the damage of tumor cells from radiation or drugs and contribute to better prognosis [16]. Such inference was supported by the present data.

CA9 is considered to be a surrogate marker of tumor hypoxia. Radiotherapy or cytotoxic drugs are less effective in hypoxic environment, thus an increased expression of CA9 may contribute for worse prognosis. Such inference is supported by the present study. By contrast, an increased expression of CA9 had a protective effect for operable squamous cell lung cancer [2] that may illustrate possible differences in prognostic significance of a given gene in surgical and non-surgical series.

ERBB3 encodes a member of the epidermal growth factor receptor (EGFR) family of receptor tyrosine kinases. High expression of this gene appeared correlated with specific clinic-pathological features of Japanese lung cancer [15]. Most importantly ERBB3 was over-expressed in non-smoking females with mutated EGFR, i.e. in a group with favorable prognostic features. While no data on EGFR mutations were available in the present series, over-expression of ERBB3 was more common in females with adenocarcinoma, supporting, thus, results of the Japanese study. Because the prognosis in females was better, over-expression of ERBB3 had a protective (but not independent) effect on survival in the present series. Interestingly, an opposite effect of ERBB3 over-expression (increased hazard of death) was revealed in the surgical study [1]. This may, again, illustrate possible differences in prognostic significance of a given gene in surgical and non-surgical series.

STAT1 is involved in up-regulating genes, leading to an increased expression of Interferon Stimulated Genes. Over-expression of STAT1 was related to better prognosis in surgical series [1], but not in the present study. The present analysis confirmed, indeed, that STAT1 over-expression was related to over-expression of the other genes (including all 6 reference genes), but the net-effect of such relationship was a trend for a protective effect of STAT1. Such outcome can be explained by strong correlation of STAT1 and CA9 apparent in the present data.

At last, FN1 is known for its role in cell adhesion, growth, migration and differentiation. Its over-expression was related to stimulation of non-small cell lung cancer growth through activation of Akt/mTOR/S6 kinase and inactivation of LKB1/AMP-activated protein kinase signal pathway [17]. Over-expression of FN1 was related to worse prognosis in the present series, but it did not appear as a useful marker in the present analysis due to correlation with clinical stage.

The prognostic score proposed requires validation using an independent dataset. We note, however, that contrary to the studies that seek for the new markers, we only explored the markers known to have a possible prognostic value in postoperative setting. One may consider, thus, that it was the present study that validated the markers provided by the other authors in a dataset that included the patients in advanced stage of disease. Testing the prognostic score prospectively in more homogenous patient groups is our future goal. Also, testing the other genes will, likely, contribute for improvement of the proposed simplistic score.

In summary, we conclude that the analysis of gene expression profiles based on bronchoscopy obtained samples appeared feasible, but the methodology that was used demands further optimization. Two-gene signature proposed for advanced inoperable NSCLC may appear useful, particularly in clinically homogeneous groups.
